# Ecological characterisation and infection of Anophelines (Diptera: Culicidae) of the Atlantic Forest in the southeast of Brazil over a 10 year period: has the behaviour of the autochthonous malaria vector changed?

**DOI:** 10.1590/0074-02760170225

**Published:** 2018-02

**Authors:** Julyana Cerqueira Buery, Helder Ricas Rezende, Licia Natal, Leonardo Santana da Silva, Regiane Maria Tironi de Menezes, Blima Fux, Rosely dos Santos Malafronte, Aloisio Falqueto, Crispim Cerutti

**Affiliations:** 1 Universidade Federal do Espírito Santo, Unidade de Medicina Tropical, Vitória, ES, Brasil; 2 Secretaria de Estado da Saúde do Espírito Santo, Vitória, ES, Brasil; 3 Universidade de São Paulo, Instituto de Medicina Tropical de São Paulo, São Paulo, SP, Brasil; 4 Superintendência de Controle de Endemias, São Paulo, SP, Brasil

**Keywords:** malária, Plasmodium vivax, Plasmodium simium, Anopheles

## Abstract

**BACKGROUND:**

In southeastern Brazil, autochthonous cases of malaria can be found near Atlantic Forest fragments. Because the transmission cycle has not been completely clarified, the behaviour of the possible vectors in those regions must be observed. A study concerning the entomological aspects and natural infection of anophelines (Diptera: Culicidae) captured in the municipalities of the mountainous region of Espírito Santo state was performed in 2004 and 2005. Similarly, between 2014 and 2015, 12 monthly collections were performed at the same area of the study mentioned above.

**METHODS:**

Center for Disease Control (CDC) light traps with CO2 were set in open areas, at the edge and inside of the forest (canopy and ground), whereas Shannon traps were set on the edge.

**FINDINGS:**

A total of 1,414 anophelines were collected from 13 species. *Anopheles (Kerteszia) cruzii* Dyar and Knab remained the most frequently captured species in the CDC traps set in the forest canopy, as well as being the vector with the highest prevalence of *Plasmodium vivax/simium* infection, according to molecular polymerase chain reaction techniques.

**CONCLUSIONS:**

*P. vivax/simium* was found only in abdomens of the mosquitoes of the subgenus *Nyssorhynchus*, weakening the hypothesis that this subgenus also plays a role in malaria transmission in this specific region.

While highly prevalent in the Amazon Region, malaria also remains residually in the Atlantic Forest regions of southern and southeastern Brazil. In these regions, the disease is known as bromeliad malaria, since the vectors of the genus *Anopheles* reproduce in the tanks of Bromeliaceae, plants typical of this biome (Downs & Pittendrigh 1946). The presence of a few autochthonous human cases and asymptomatic infections, the spatial distance between the cases, and the low parasitaemia by microscopy provide evidence in opposition to the traditional transmission chain. It is believed that the cycle of *Plasmodium* species in the Brazilian Atlantic Forest is maintained by reservoirs of the parasite, in the forest or in the rural population, comprised of both simians and humans, asymptomatic or not; and studies have been conducted to test the validity of these assumptions (Curado et al. 1997, Duarte et al. 2006, Cerutti Jr et al. 2007, Meneguzzi et al. 2009, Rezende et al. 2009). Regarding the vector, in the state of Espírito Santo, Brazil, 26 species of *Anopheles* have been identified, indicating that this genus is commonly found in the region (Coutinho 1947, Andrade & Brandão 1957, Ferreira 1964, Natal et al. 2007, Sallum et al. 2008, Meneguzzi et al. 2009, Rezende et al. 2009, Silva et al. 2013). The vectors of greatest vectorial capacity and competence belong to the subgenus *Kerteszia*, mainly *Anopheles* (*Kerteszia*) *cruzii*, in the southern and southeastern Brazilian states (Meneguzzi et al. 2009). However, the species of anophelines involved in the dynamics of malaria transmission outside the Amazon Region vary according to environmental and epidemiological conditions (de Pina-Costa et al. 2014). Autochthonous cases of extra-Amazonian malaria, mostly caused by *P. vivax*, are found in moderately mountainous regions and are associated with agricultural activities performed by young men near the forest (Cerutti Jr et al. 2007). In addition to that, however, there is one more malaria transmission site in Espírito Santo. The northern region of the state is a frequent scenario of disease outbreaks. These outbreaks come from introducers from the Amazon Region. Records of *P. falciparum* autochthonous malaria in Espírito Santo are related to these outbreaks. In the cases detected at the site of each outbreak, although they are also called autochthonous cases by epidemiological definition, the disease is brought in from the outside, unlike what is observed in the Atlantic Forest. Therefore, at our present level of knowledge, it is not possible to recognise the occurrence of symptomatic malaria caused by *P. falciparum* in an Atlantic Forest environment, as it is for cases caused by *P. vivax*. States such as São Paulo, Santa Catarina and Espírito Santo are covered by dense Atlantic Forest regions (IESB 2007). Among them, Espírito Santo has recorded the largest number of bromeliad-malaria cases in recent years. The biome in question is very humid, with abundant rainfall and vegetation that favours the reproduction of anophelines. In addition, bromeliad-malaria is beginning to be used as a biological marker of human activities within these forests (Gomes et al. 1985, Rezende et al. 2013). Rezende et al. (2009) found anopheline behaviour similar to that expected, based on the literature, in Valsugana Velha, Santa Teresa municipality: a high prevalence of *A.* (*K.*) *cruzii* was reported in a mountainous region of the Atlantic Forest, in an endemic area of the disease in the state of Espírito Santo. However, regarding the hypothesis of a typical transmission model of zoonosis in this scenario, this model would require a stable and constant vectorial behaviour. Such behaviour, therefore, must be repeatedly assessed in order to establish transmission characteristics over the course of years.

## MATERIALS AND METHODS

**Fig. 1:**
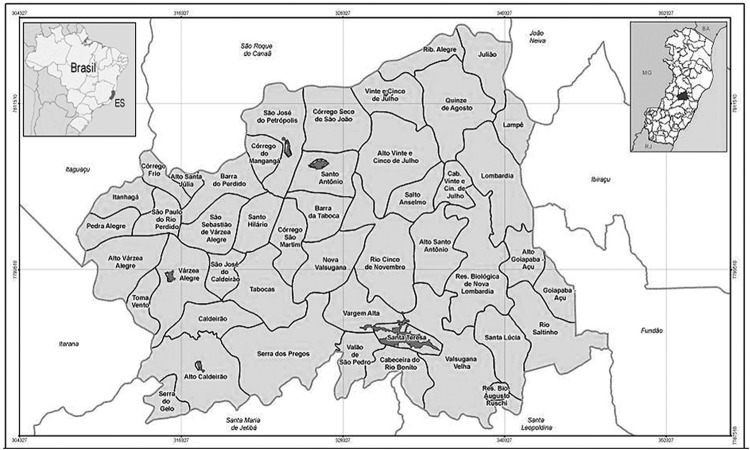
political map of the municipality of Santa Teresa, Espírito Santo, Brazil, highlighting the rural community where the collections were performed.

Source: Instituto Jones dos Santos Neves, 2013.

*Description of the study site* - The study was performed in the rural area of the municipality of Santa Teresa, which is located approximately 78 km from Vitória, in the state of Espírito Santo (ES), Brazil. The permanent trapping station is in Valsugana Velha (19°57′58.4″ S, 40°34′45.2″ W), where cases of residual malaria from Atlantic Forest systems were recorded in the study by Cerutti Jr. et al. (2007) (Fig. 1).

*Collection of anophelines* - Hourly collections of anophelines were conducted at a permanent trapping station located in the bromeliad malaria transmission area. These collections were performed one day each month for one year, from June 2014 to May 2015, totalling 12 collections. Two capture methods were used: (i) Center for Disease Control (CDC) light traps with CO_2_ (Gomes et al. 1985) that were set in open areas (peridomiciliary environment = PD), at the edge of the forest (canopy and ground) and inside the forest (canopy and ground); and (ii) Shannon traps (Bustamante & Pires 1951), set at the edge of the forest.

The five CDC traps were set simultaneously, two of them at 15 m from the ground in the canopy (on the edge and inside the forest), two at one metre from the ground (at the edge and inside the forest), and one at the border between the forest and the area close to dwellings. The collections lasted for 12 h, with the traps placed at night (6:00 PM) and removed in the morning (6:00 AM). For the Shannon traps, the insects were captured during the first 4 h after sunset (6:00 PM to 10:00 PM) each month.

*Storage and identification of insects* - The specimens were stored in tubes containing isopropanol and later identified using the identification keys proposed by Consoli and Lourenço-de-Oliveira (1994). Identification was performed by a team from the Entomology and Malacology Centre of Espírito Santo (Núcleo de Entomologia e Malacologia do Espírito Santo - NEMES/ES).

*Molecular techniques* - The DNA for the detection of *Plasmodium* species was obtained from the thorax, abdomen or entire mosquito of the mosquito groupings in pools (maximum of 10 samples/pool), depending on the subgenus of the specimens. Those of the subgenus *Nyssorhynchus* were sectioned (separated pools of thoraxes and abdomens), whereas those of the subgenus *Kerteszia* were processed in whole. The same extraction kit (DNeasy Blood & Tissue Kit, Qiagen, Germany) was used, following the manufacturer's instructions. Each pool included females of the same species, collected in the same type of trap, on the same date. The presence of *P. vivax/simium* or *P. malariae* in the pools was detected using the nestedpolymerase chain reaction (PCR) protocol described by Kimura et al. (1997) and modified by Win et al. (2002). The target was the gene encoding the 18S ribosomal subunit. The amplification products were analysed by electrophoresis in 2% agarose gel under ultraviolet light. In positive cases, 100-bp fragments were amplified.

*Statistical analysis* - To determine the importance and distribution of the various *Anopheles* species, analyses of diversity, dominance and abundance were performed using Shannon's diversity index (H′) and Simpson's dominance index (D). In the various comparisons, the level of significance was set at 5%. Bivariate Spearman correlation calculations were used to determine the relationship between anopheline capture, temperature and rainfall [data provided by the Capixaba Institute of Research, Technical Assistance and Rural Extension (Instituto Capixaba de Pesquisa, Assistência Técnica e Extensão Rural - INCAPER)], also at a level of 5%.

*Ethical standards* - During the collection process, no harm was inflicted to the environment. The team members wore long clothing, gloves and hats with head nets to avoid anopheline mosquito bites. Boots were also used to prevent bites by venomous animals. A license to capture arthropod insects was obtained via the SISBIO/Chico Mendes Institute (ICMBio/IBAMA/MMA) under No. 19227-1.

## RESULTS

A total of 1,414 specimens were captured, resulting in a set of 13 species. The largest number of specimens was collected in April 2015 (341), and the smallest number of specimens in March 2015 (5). In all collections, there was a predominance of *A*. (*K.*) *cruzii*, totalling 1,044 of the 1,414 mosquitoes captured (Table I). The trap in which most mosquitoes were captured was the CO_2_-baited CDC trap placed in the canopy, in which *A.* (*K.*) *cruzii* was also predominant (Fig. 3).

**TABLE I:** Percentage of anopheline species found between June 2014 and May 2015 at the permanent trapping station in Santa Teresa, Espírito Santo, Brazil

Species	Number	(%)
*Anopheles (Kerteszia) cruzii*	1044	73.8
*A. (Nyssorhynchus) strodei*	103	7.3
*A. (N.) triannulatus*	84	6.0
*A. (N.) evansae*	52	3.7
*A. (K.) homunculus*	39	2.7
*A. (N.) galvaoi*	29	2.0
*A. (N.) albitarsis*	17	1.2
*A. (N.) parvus*	07	0.5
*A. (A.) mediopunctatus*	05	0.4
*A. (N.) rangeli*	03	0.2
*A. (N.) lanei*	01	0.1
TOTAL	1414	100

**Fig. 2:**
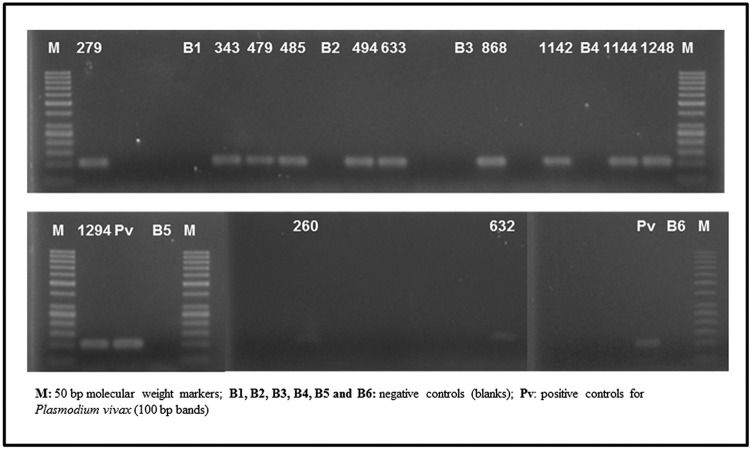
ethidium bromide-stained agarose gel, photographed using the Alpha Imager software, with 13 samples of pools of *Plasmodium vivax/simium*-positive anopheline females.

**Fig. 3:**
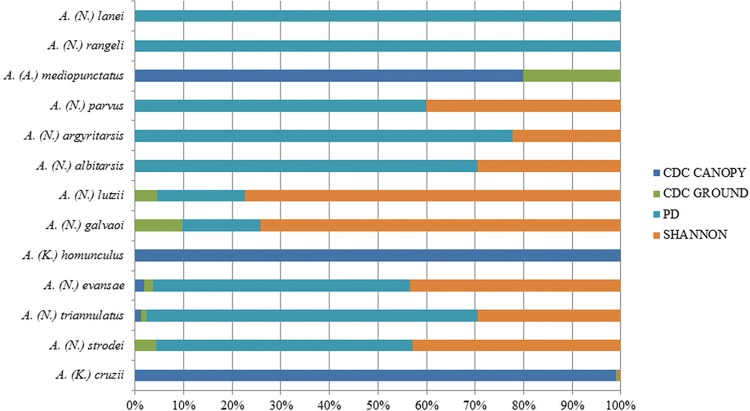
percentage of species captured per trap, from June 2014 to May 2015, at the permanent trapping station in Santa Teresa, Espírito Santo, Brazil.

**Fig. 4:**
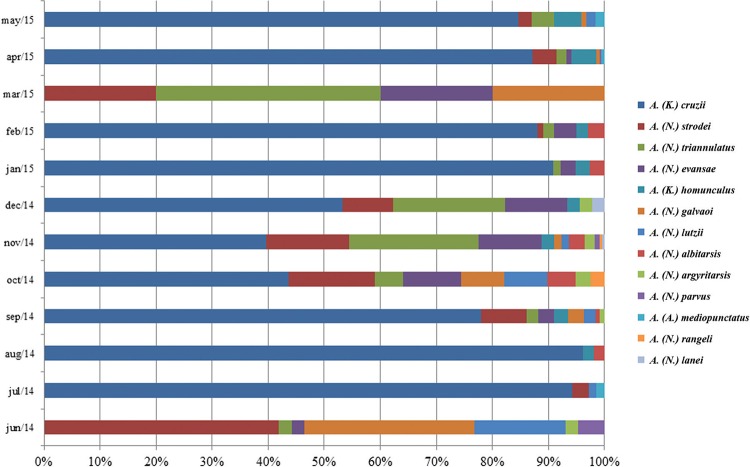
species captured each collection month, from June 2014 to May 2015, at the permanent trapping station of Santa Teresa, Espírito Santo, Brazil.

*Climate* - The months of November 2014, September 2014, and April 2015 showed the highest capture frequencies (Fig. 4). There was no simultaneous capture of all species in the monthly collections, and all species were absent for at least 1 month during the total collection period. *A.* (*K.*) *cruzii* was not captured only in June 2014 and March 2015 (Fig. 4). In fact, those were the only months in which *A.* (*K.*) *cruzii* ceased to be the predominant species and was replaced with *A.* (*Nyssorhynchus*) *strodei* and *A.* (*Nyssorhynchus*) *triannulatus*, respectively.

**Fig. 5:**
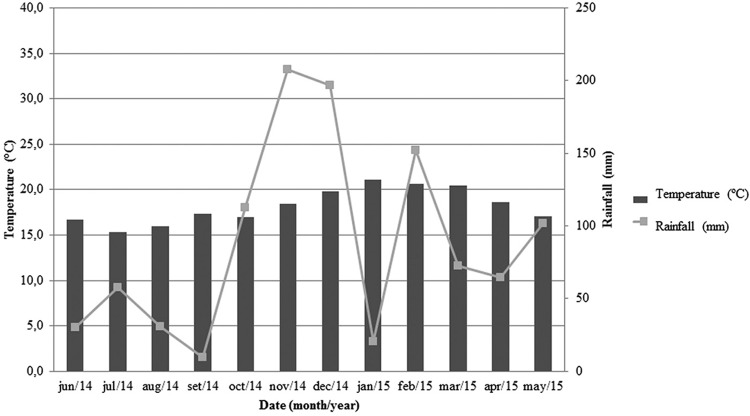
temperature and rainfall data in Santa Teresa, Espírito Santo, Brazil, between June 2014 and May 2015.

A negative trend was observed for the correlation between the number of anophelines of the subgenus *Nyssorhynchus* and the temperature, although without statistical significance (r = - 0.04, p = 0.89). Additionally, a positive trend was observed for the correlation between the number of anophelines and rainfall (r = 0.17, p = 0.59) but also with no statistical significance. For the subgenus *Kerteszia*, the correlation between capture frequency and temperature (r = -0.004, p = 0.99) or rainfall (r = -0.13, p = 0.68) revealed negative coefficients but with no statistical significance. The monthly average temperature and the rainfall in Valsugana Velha are shown in Fig. 5.

*Spatial distribution* - *A.* (*N.*) *strodei* and *A.* (*N.*) *triannulatus* were more frequently captured in the Shannon traps, and *A.* (*K.*) *cruzii* was captured more frequently in the CO_2_-baited CDC traps (Fig. 3) located in the tree canopy. Simpson's dominance index (D) reveals that dominance in the Shannon trap (D1 = 0.227) was greater than the dominance of individuals collected in the CO_2_-baited CDC trap (D2 = 0.172) (p < 0.02), both at the forest edge.

Based on Shannon's diversity index (H′), the diversity of anophelines collected in the Shannon trap (H′2 = 1.866) was higher compared to that of the anophelines collected in the CDC trap at the forest edge, near the dwellings (H′1 = 1.734) (p = 0.004). *A.* (*N.*) *strodei* was captured in larger numbers at forest edge areas, followed by *A.* (*N.*) *triannulatus*. *A.* (*N.*) *lanei* and *A.* (*N.*) *rangeli* were captured only in the CO_2_-baited CDC trap located near the dwellings, whereas *A.* (*K.*) *homunculus* was only captured in the CDC traps set in the tree canopy.

**Fig. 6:**
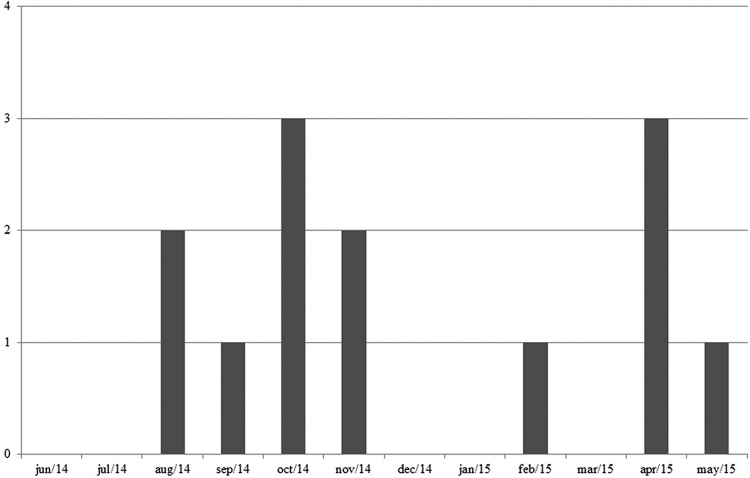
chronological distribution of *Plasmodium vivax/simium*-infected anopheline females captured in the permanent trapping station in Santa Teresa, Espírito Santo, Brazil.

*Presence of Plasmodium in the anophelines (* Table II *)* - Of the total pool of specimens investigated, 13 were positive for *P. vivax/simium* (Fig. 2). Of these, 10 belonged to the species *A. cruzii*, and three were abdomens that belonged to specimens of the subgenus *Nyssorhynchus*. As shown in Table II, most of the mosquitoes with *Plasmodium* DNA were *A. cruzii*, captured in CO_2_-baited CDC traps located in the tree canopy. Fig. 6 shows the monthly distribution of captured mosquitoes in which *P. vivax/simium* was found. *P. malariae* infection was not detected.

**TABLE II:** Species, traps and collection dates for *Plasmodium vivax/simium*-positive mosquitoes at the permanent trapping station in Santa Teresa, Espírito Santo, Brazil

Sample	Species	Trap	Date
260*a* *	*A. (Nyssorhynchus) lutzii*	Shannon	September 2014
279	*A. (Kerteszia) cruzii*	CDC canopy	August 2014
343	*A. (K.) cruzii*	CDC canopy	August 2014
479	*A. (K.) cruzii*	CDC canopy	October 2014
485	*A. (K.) cruzii*	CDC canopy	October 2014
494*a* *	*A. (N.) evansae*	Shannon	October 2014
632	*A. (K.) cruzii*	CDC ground	November 2014
633	*A. (K.) cruzii*	CDC canopy	November 2014
868	*A. (K.) cruzii*	CDC canopy	February 2015
1142	*A. (K.) cruzii*	CDC canopy	April 2015
1144	*A. (K.) cruzii*	CDC canopy	April 2015
1248	*A. (K.) cruzii*	CDC canopy	April 2015
1294*a* *	*A. (N.) strodei*	CDC near dwellings	May 2015

* *a*: abdomen.

## DISCUSSION

Evidence of residual bromeliad malaria in the south and southeast Brazilian Atlantic Forest regions arose after control measures implemented by the government had eliminated transmission in flat areas exploited by humans (Barata 1998, MS 2015). As residual foci remained in the eradication areas, bromeliad malaria, or malaria of the Atlantic Forest systems, began to be studied with more intensity in the 1980s and 1990s (Duarte et al. 2013). With a more consistent understanding of vector ecology and outbreak epidemiology, more evidence has emerged in favour of behaviour consistent with a zoonosis. The zoonosis hypothesis presupposes a concomitant existence of simian malaria. Such an existence has been verified in the past (Duarte et al. 2008), but its prospective evaluation demands extremely complex logistics. Therefore, the evaluation of vector behaviour favourable to enzootic transmission is more feasible and may provide indirect evidence of the presence of the infection among monkeys and of its source of occurrence in human cases. Given the requirement of stable and constant vector behaviour for the maintenance of simian malaria, follow-up studies of the endemic regions are necessary. In addition, according to Marrelli et al. (2007), the entire Atlantic Forest territory that survived deforestation must be carefully monitored due to environmental changes and the possibility of maintaining the infection cycle given the high number of asymptomatic individuals who can act as reservoirs of the parasite in those regions.

In the present study, *A.* (*K.*) *cruzii* still prevailed as the main vector found at the Valsugana Velha trapping station. Between 2004 and 2005 (Rezende et al. 2009), 61.2% of the 2,290 anophelines collected belonged to this species. In 2014 and 2015, 73.8% of the 1,414 anophelines captured were *A.* (*K.*) *cruzii*. Therefore, an increase in the proportion of the main vector of bromeliad malaria was observed in the local fauna. Older studies such as those of Deane (1988), and more recent studies such as those of Duarte et al. (2013), Neves et al. (2013) and Kirchgatter et al. (2014) have previously demonstrated the magnitude of the presence of this species in Atlantic Forest regions with preserved native forest.

The distribution of *A.* (*K.*) *cruzii* when moving from the inside to the edge of the forest has also remained the same over time. As Rezende et al. (2009) described in 2004 and 2005, the anophelines of this species appeared mostly in the tree canopy inside the forest, whereas the numbers decreased drastically in the traps that were set closer to the forest edge and the dwellings. The anopheline fauna was dominated by species such as *A.* (*N.*) *strodei* and *A.* (*N.*) *triannulatus* closer to human-occupied areas. In São Paulo, between 2009 and 2011, this trend was also detected when comparing anthropic and wild areas in the town of Parelheiros. There, 438 *A.* (*K.*) *cruzii* were recorded in a certain anthropic area and 4,832 in the wild area (Duarte et al. 2013).

The absolute predominance of *A.* (*K.*) *cruzii* in the canopy, observed in the present study, indicates a nonsynanthropic behaviour (Forattini et al. 1990) and reinforces its role in the transmission of simian malaria. However, despite being present mostly in the canopy, some specimens were found in the CO_2_-baited CDC traps set in the ground and in the Shannon traps, which points to the possibility of these vectors descending from their preferential site in the canopy, at which time they could incidentally transmit the parasites to humans. The presence of these specimens reinforces the hypothesis that bromeliad malaria is maintained in the region by means of simian infection. The fact that humans are in the forest and that *A.* (*K.*) *cruzii* comes down from the canopy to feed creates the conditions for *Plasmodium* specimens originating from the simians in the canopy to infect humans.

The morphometric diagnosis studies of Lorenz et al. (2012) showed a differentiation between *A.* (*K.*) *cruzii* and *A.* (*K.*) *homunculus* not predicted in the study of Rezende et al. (2009). However, only 39 *A.* (*K.*) *homunculus* were captured during the collection period between 2014 and 2015, against 1,045 duly identified *A.* (*K.*) *cruzii*.

One of the limitations of this study was that species identification was made only on a morphological basis. To minimise the possibility of misidentification, we improved the accuracy through evaluation by experienced entomologists and through a double identification performed in cases of doubt.

Unexpectedly, the season during which most mosquitoes were captured was not the summer. Interestingly, the seasons with milder weather had the largest capture rates. For example, the most successful captures occurred in the months of September 2014 and April 2015. This result corroborates the study of Rezende et al. (2009), who suggested the adaptation of anophelines to mild environments. There was also a high number of specimens collected in November 2014. The mean rainfall at that time was the highest of that year (mean of 207.6 mm/month), and the temperature was mild on collection day (ranging from 19.7 to 21.3°C). That month, the “rain” factor may have triggered an increase in the mosquito population.

In this study, 13 pools of mosquitoes were positive for *P. vivax/simium*. In 2004 and 2005, 10 pools of anophelines infected by the same species of the parasite were obtained (Rezende et al. 2009). However, unlike 10 years ago, the PCR reactions for the 2014 and 2015 collections did not show infection in the thorax of mosquitoes of the subgenus *Nyssorhynchus*. The infective form should reach the salivary gland of the anopheline for infection to occur. Thus, the separation into thorax and abdomen during the experiments reinforces evidence of the participation of other vector species in the transmission chain. Because *Kerteszia* is a subgenus of a known vector, there was no separation of the body to perform the experiments, as occurred in mosquitoes of the subgenus *Nyssorhynchus*, to assess the possibility that these mosquitoes participate in the transmission chain. Since no infection was detected in the *Nyssorhynchus* specimens thoraxes, unlike 10 years ago, *Nyssorhynchus* species may have stopped playing the role of secondary vector and *A.* (*K.*) *cruzii* may currently be the only bromeliad malaria vector in this area. The progressive exploitation of the rural and forest environment by the local inhabitants may have led to greater spatial distances between the transmission events and the anthropic environment, where *Nyssorhynchus* species have greater dominance. In these conditions, given the *A.* (*K.*) *cruzii* dominance, *Nyssorhynchus* species does not have the opportunity to become infected. However, humans venture into the forest to clean the river springs or to gather firewood and thus acquire the disease. Once they return to their homes, they likely transmit the parasites to the *Nyssorhynchus* specimens near their dwellings.

Infected *A.* (*K.*) *cruzii* were collected in CO_2_-baited CDC traps in the tree canopy inside the forest and in one CO_2_-baited CDC trap located near the ground, at a height of one metre. This fact reinforces the idea that infections can occur in both habitats as a result of the acrodendrophilic behaviour with eventual descent to lower heights, where they can feed on non-usual hosts. This fact also reinforces the possibility of the disease being a zoonosis in regions such as Valsugana Velha, in Santa Teresa, ES.

Regarding the Shannon's dominance index (H′), there was greater dominance in the Shannon trap than in the CO_2_-baited CDC trap, both at the forest edge.

Regarding Simpson's diversity index (D), the diversity of anophelines collected with the Shannon trap was also higher than that observed in the CO_2_-baited CDC trap in the same habitat. The placement of the trap near the body of water, where there were *Nyssorhynchus* species breeding sites, would justify the greater diversity.

These data corroborate a study conducted in the same region and published in 2013 (Rezende et al. 2013), when greater dominance and diversity of anophelines were observed in an anthropic environment with the presence of malaria. These findings support the role of human occupation in the determination of both anopheline distribution and behaviour, since both indices show higher values in collections performed in an environment closer to human dwellings.

The study shows that there was little change in vector behaviour in the region studied. *A.* (*K.*) *cruzii* remains the mosquito with the higher evidence of natural infection in Valsugana Velha, and mosquitoes of the subgenus *Nyssorhynchus* do not seem to participate in the transmission chain. The acrodendrophilic behaviour of *A. (K.) cruzii*, particularly those infected, strengthens the hypothesis that the presence of *P. vivax/simium* in these specimens arises from blood feeding in animals that live in the tree canopy, such as simians.

## References

[B1] Andrade RM, Brandão H (1957). Contribuição para o conhecimento da fauna de anofelinos do estado do Espírito Santo: área de distribuição e incidência das espécies por cidades, vilas e povoados. Rev Bras Malariol Doenças Trop.

[B2] Barata RB (1998). Malária e seu controle.

[B3] Bustamante FM, Pires WM (1951). Shannon dawn trap: its use in the verification of the durability of residual toxic effects of insecticides. Folha Med.

[B4] Cerutti C, Boulos M, Coutinho AF, Hatab MCLD, Falqueto A, Rezende HR (2007). Epidemiologic aspects of the malaria transmission cycle in an area of very low incidence in Brazil. Malar J.

[B5] Consoli RAGB, Lourenço-de-Oliveira R (1994). Principais mosquitos de importância sanitária no Brasil. Cad Saude Publica.

[B6] Coutinho JO (1947). Contribuição para o estudo da distribuição geográfica dos anofelinos do Brasil.

[B7] Curado I, Duarte AMRC, Lal AA, Oliveira SG, Kloetzel JK (1997). Antibodies anti bloodstream and circumsporozoite antigens (*Plasmodium vivax* and *Plasmodium malariae/P. brasilianum*) in areas of very low malaria endemicity in Brazil. Mem Inst Oswaldo Cruz.

[B8] de Pina-Costa A, Brasil P, Di Santi SM, de Araujo MP, Suárez-Mutis MC, Santelli ACFS (2014). Malaria in Brazil: what happens outside the Amazonian endemic region. Mem Inst Oswaldo Cruz.

[B9] Deane LM (1988). Malaria studies and control in Brazil. Am J Trop Med Hyg.

[B10] Downs WG, Pittendrigh CS (1946). Bromelian malaria in Trinidad, British West Indies. Am J Trop Med.

[B11] Duarte AM, Malafronte RS, Cerutti C, Curado I, Paiva BR, Maeda AY (2008). Natural *Plasmodium* infections in Brazilian wild monkeys: reservoirs for human infections?. Acta Trop.

[B12] Duarte AM, Pereira DM, de Paula MB, Fernandes A, Urbinatti PR, Ribeiro AF (2013). Natural infection in anopheline species and its implications for autochthonous malaria in the Atlantic Forest in Brazil. Parasit Vectors.

[B13] Duarte AMRC, Porto MAL, Curado I, Malafronte RS, Hoffman EHE, Oliveira SG (2006). Widespread occurrence of antibodies against circumsporozoite protein and against blood forms of *Plasmodium vivax*, *P*. *falciparum* and *P*. *malariae* in Brazilian wild monkeys. Int J Primatol.

[B14] Ferreira E (1964). Distribuição geográfica dos anofelinos no Brasil e sua relação com o estado atual da erradicação da malária. Rev Bras Malariol Doenças Trop.

[B15] Forattini OP, Kakitani I, Massad E, Marucc D (1990). Studies on mosquitoes (Diptera: Culicidae) and anthropic environment. 11 - biting activity and blood-seeking parity of *Anopheles* (Kerteszia) in SouthEastern Brazil. Rev Saude Publica.

[B16] Gomes AC, Rabello EX, Natal D (1985). A new collecting chamber for a CDC-miniature trap. Rev Saude Publica.

[B17] IESB - Instituto de Estudos Socioambientais do Sul da Bahia (2007). Projeto de conservação e utilização sustentável da diversidade biológica brasileira - PROBIO. Levantamento da cobertura vegetal nativa do bioma Mata Atlântica.

[B18] Kimura M, Kaneco O, Liuc Q, Zhouc M, Kawamotoc F, Watayad Y (1997). Identification of the four species of human malaria parasites by nested PCR that targets variant sequences in the small subunit rRNA gene. Parasitol Int.

[B19] Kirchgatter K, Tubaki RM, Malafronte RM, Alves IC, Lima GFMC, Guimarães LO (2014). *Anopheles (Kerteszia) cruzii* (Diptera: Culicidae) in peridomiciliary area during asymptomatic malaria transmission in the Atlantic Forest: molecular identification of blood-meal sources indicates humans as primary intermediate hosts. Rev Inst Med Trop Sao Paulo.

[B20] Lorenz C, Marques T, Sallum MA, Suesdek L (2012). Morphometrical diagnosis of the malaria vectors *Anopheles cruzii*, *An. homunculus* and *An. bellator*. Parasit Vectors.

[B21] Marrelli MT, Malafronte RS, Sallum MAM, Natal D (2007). Kerteszia subgenus of *Anopheles* associated with the Brazilian Atlantic Rainforest: current knowledge and future challenges. Malar J.

[B22] Meneguzzi VC, dos Santos CB, Pinto IS, Feitoza LR, Feitoza HN, Falqueto A (2009). Use of geoprocessing to define malaria risk areas and evaluation of the vectorial importance of anopheline mosquitoes (Diptera: Culicidae) in Espírito Santo, Brazil. Mem Inst Oswaldo Cruz.

[B23] MS - Ministério da Saúde (2015). Ministério da Saúde lança Plano de Eliminação da malária no Brasil.

[B24] Natal D, Urbinatti PR, Malafronte RS, Rezende HR, Cerutti C, Sallum MAM (2007). First record of *Anopheles* (*Anopheles*) *costai* Fonseca & Ramos, 1939 in Espírito Santo State. Rev Inst Med Trop Sao Paulo.

[B25] Neves A, Urbinatti PR, Malafronte RS, Fernandes A, Paganini WS, Natal D (2013). Malaria outside the Amazon Region: natural *Plasmodium* infection in anophelines collected near an indigenous village in the Vale do Rio Branco, Itanhaém, SP, Brazil. Acta Trop.

[B26] Rezende HR, Falqueto A, Urbinatti PR, de Menezes RM, Natal D, Cerutti C (2013). Comparative study of distribution of anopheline vectors (Diptera: Culicidae) in areas with and without malaria transmission in the highlands of an extra-Amazonian region in Brazil. J Med Entomol.

[B27] Rezende HR, Soares RM, Cerutti C, Alves IC, Natal D, Urbinatti PR (2009). Entomological characterization and natural infection of anophelines in an area of the Atlantic Forest with autochthonous malaria cases in mountainous region of Espírito Santo state, Brazil. Neotrop Entomol.

[B28] Sallum MAM, Urbinatti PR, Malafronte RS, Rezende HR, Cerutti C, Natal D (2008). Primeiro registro de *Anopheles* (*Kerteszia*) *homunculus* Komp (Diptera, Culicidae) no estado do Espírito Santo, Brasil. Rev Bras Entomol.

[B29] Silva KS, Pinto IS, Leite GR, Virgens TM, Santos CB, Falqueto A (2013). Ecology of Anopheline mosquitoes (Diptera: Culicidae) in the Central Atlantic Forest biodiversity corridor, southeastern Brazil. J Med Entomol.

[B30] Win TT, Lin K, Mizuno S, Zhou M, Liu Q, Ferreira MU (2002). Wide distribution of *Plasmodium ovale* in Myanmar. Trop Med Int Health.

